# From Measured Accuracy to Patient Outcome: Navigation in Total Hip Arthroplasty and Molecular Diagnosis of Periprosthetic Joint Infection: A Narrative Review

**DOI:** 10.3390/diagnostics16203285

**Published:** 2026-10-10

**Authors:** Vladislav Andonov, Vensan Velchov, Yasen Nikolov, Nikolay Cherkezov, Dimitar Tenev, Michail Dimitrov, Tsvetan Tsenkov, Nikolay Dimitrov

**Affiliations:** Specialized University Hospital for Active Treatment in Orthopaedics “Prof. Boycho Boychev”, Medical University of Sofia, 1614 Sofia, Bulgaria

**Keywords:** computer-assisted navigation, total hip arthroplasty, periprosthetic joint infection, osteomyelitis, metagenomic next-generation sequencing, single nucleotide polymorphism, diagnostic accuracy, administrative claims data, morbid obesity, case definition

## Abstract

**Background:** Total hip arthroplasty (THA) and the diagnosis of periprosthetic joint infection (PJI) have each acquired a technology that measures with high precision: intraoperative navigation, which reports component position to within a few degrees, and molecular methods, which identify the infecting organism and the host response at genomic resolution. **Objective:** To establish whether the measured accuracy has been shown to reach the patient as a clinical outcome. **Methods:** This is a narrative review, reported according to SANRA. MEDLINE, Scopus, and Web of Science databases were searched from inception to 31 August 2026. English-language studies in humans were eligible if they reported positional or diagnostic accuracy, a patient-level clinical or economic outcome after THA or after treatment of PJI or osteomyelitis, or a factor modifying the relation between the two. Animal, in vitro, and cadaveric studies, conference abstracts, and reports without extractable data were excluded. Forty studies were included. No pooled estimate was calculated, and no new statistical analysis was performed. **Results:** Navigation reduced positional outliers consistently, but in two matched claims-based cohorts of patients with morbid obesity (4499 and 5835 patients), it was not associated with lower rates of PJI, dislocation, periprosthetic fracture, mechanical loosening, or revision within two years. Candidate immunogenetic variants in osteomyelitis have never been tested against a treatment decision. Metagenomic sequencing was no more accurate than culture (sensitivity 63.2% versus 68.4%; specificity 80.0% versus 95.0%) except after antibiotic exposure. Reported infection rates and test accuracy both change with the case definition applied. **Conclusions:** In both settings, the limiting factor is the design of the studies linking measurement to outcome rather than the precision of the measurement.

## 1. Introduction

Precision and benefit are not the same measure. An intraoperative navigation system that places an acetabular component within three degrees of a preoperative plan has demonstrated precision. Whether that precision reaches the patient as fewer dislocations, fewer revisions, or better function is a separate empirical question, answered with different data and on a longer timescale.

The same separation applies to diagnosis. A sequencing platform that identifies an organism in a culture-negative joint aspirate has demonstrated analytical sensitivity. Whether the identification changes antimicrobial selection, and whether the changed selection improves eradication, are downstream questions. The two questions are often conflated.

This review addresses one question in two clinical settings: has the measured accuracy of a precision technology been shown to reach the patient as a clinical outcome? The two settings are elective primary total hip arthroplasty (THA), where the technology is intraoperative navigation, and periprosthetic joint infection (PJI) of the hip and knee together with native-bone osteomyelitis, where the technologies are host immunogenetic profiling and pathogen-side molecular diagnostics. These two settings were chosen because they are linked: PJI is a complication of the arthroplasty addressed in the first setting, and both technologies are promoted on the same argument—that a more exact measurement will produce a better result for the patient.

Four questions are addressed in turn, and each is answered against a named outcome. First, how accurately does navigation place the acetabular component in THA, with component position as the outcome (Section 3.1)? Second, is that positional accuracy associated with fewer complications and revisions after THA, with infection, dislocation, periprosthetic fracture, mechanical loosening, and revision as the outcomes (Section 3.2, Section 3.3 and Section 3.4)? Third, how accurately do host genomic and pathogen molecular methods identify and characterise bone and joint infection, with sensitivity and specificity against a defined case standard as the outcome (Section 3.5, Section 3.6 and Section 3.7)? Fourth, has either technology been shown to change a treatment decision or a patient-reported result, with infection eradication and measured function as the outcomes (Section 3.8 and Section 3.9)?

**Figure 1:**
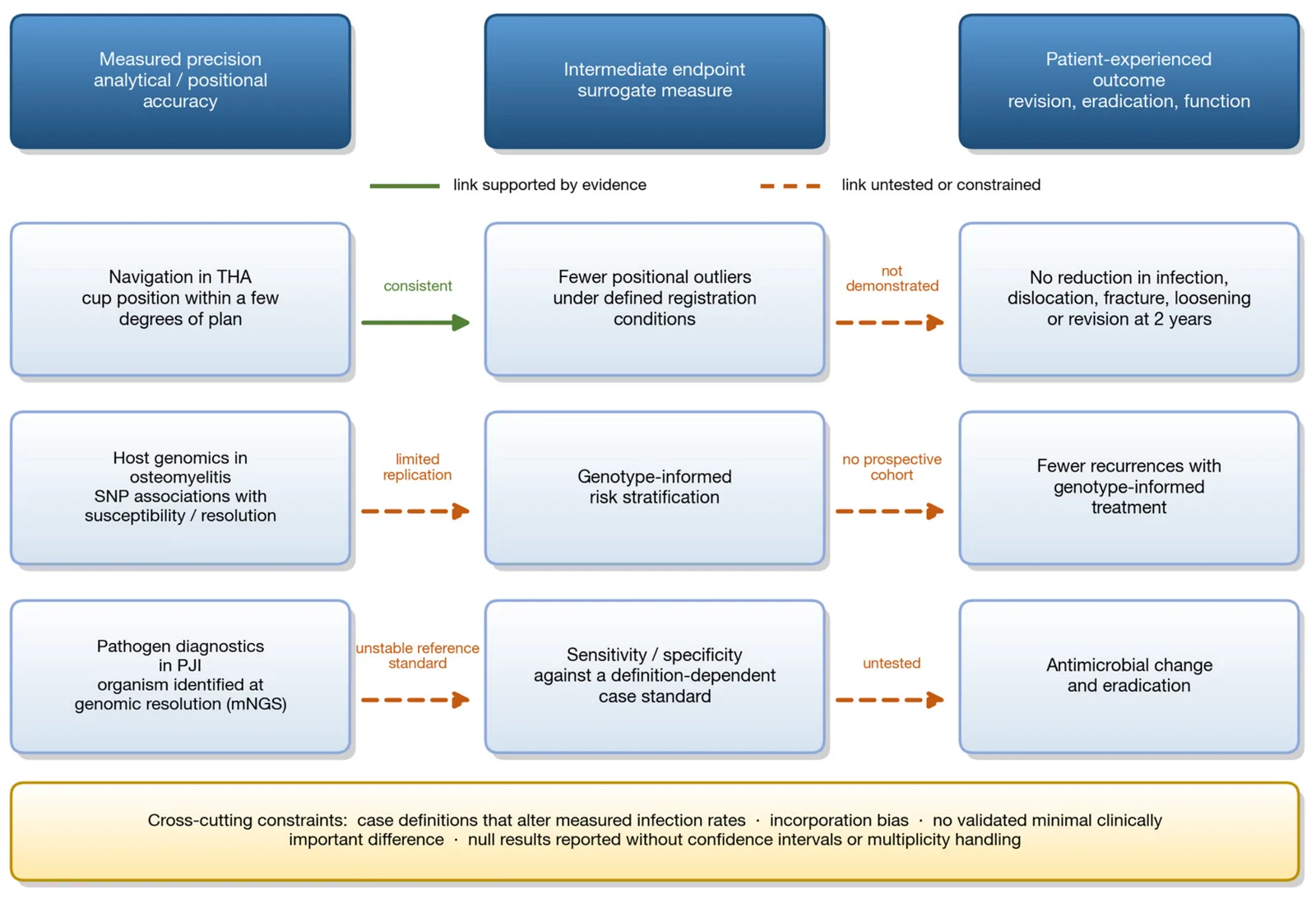
The accuracy-to-outcome evidence chain in the two clinical settings reviewed. Solid arrows denote links supported by published evidence; dashed arrows denote links that are untested or constrained by the limitation named above them. The figure is an original synthesis prepared by the authors; the evidence on which each link is classified is taken from the following sources: navigation accuracy [1,2,3,4,5,6,7,8,9]; navigation and clinical outcome [10,11]; host genomic associations in osteomyelitis [12]; genotype-informed stratification [12]; pathogen molecular diagnostics and their accuracy [13,14,15,16,17]; case definition dependence of the reference standard [18,19,20,21]; treatment decision and eradication [22]; outcome measurement [23,24,25]. THA, total hip arthroplasty; SNP, single nucleotide polymorphism; PJI, periprosthetic joint infection; mNGS, metagenomic next-generation sequencing.

The evidence is not weak in the same way in each setting. For navigation in THA, patient-level outcomes have been tested directly, and no benefit was detected in the population studied, which is evidence of the absence of a large effect in that population. For host genomics and molecular pathogen diagnostics, patient-level benefit has largely not been tested at all, which is an absence of evidence. The distinction is maintained throughout because it leads to different recommendations: the first calls for better-powered and longer studies, the second for any outcome study at all. The structure of the argument and the points at which the chain from measurement to outcome breaks are shown in Figure 1.

Two boundaries are stated at the outset. This review does not survey orthopaedic surgery as a discipline, and it does not survey the robotic arthroplasty literature, computer-assisted total knee arthroplasty, spinal navigation, fracture navigation, or trauma applications, each of which has a separate evidence base. Robotic THA and robotic total knee arthroplasty are cited only where pooled analyses of them bear directly on the size of the effect that navigation studies would need to detect. Conditions other than hip osteoarthritis treated by THA, PJI, and osteomyelitis are outside the scope and are not reviewed.

## 2. Materials and Methods

### 2.1. Design

This is a narrative review conducted according to the Scale for the Assessment of Narrative Review Articles (SANRA) [26], with selected transparency elements adopted from systematic review practice. It is not a systematic review. No pooled estimate, no meta-analysis, and no new statistical calculation of any kind were performed. Every effect estimate, confidence interval, and *p*-value reported below is reproduced as published by the original investigators and is attributed to them. Where an original report omits a confidence interval or does not state how multiplicity was handled, that omission is recorded as a limitation of that report rather than repaired by calculation here.

### 2.2. Search Strategy

MEDLINE (PubMed), Scopus and Web of Science were searched from inception to 31 August 2026. The search was structured as six strategies. Each combined a technology or measurement block with a clinical setting block using the Boolean operator AND, with controlled vocabulary (MeSH) and title/abstract free-text terms combined within each block using OR. All six were filtered to English[lang], excluded records indexed as animal and not human, and were capped at a publication date of 31 August 2026. The six strategies were as follows.

S1, navigation and robotics in THA: (Surgery, Computer-Assisted[mh]; Robotic Surgical Procedures[mh]; computer-assisted navigation; computer navigation; surgical navigation; imageless navigation; navigation system; navigated; robotic-assisted; robot-assisted; robotic arm) AND (Arthroplasty, Replacement, Hip[mh]; total hip arthroplasty; total hip replacement; hip arthroplasty; hip replacement; THA).

S2, THA in obesity and in administrative claims data: the THA block above AND ((Obesity[mh]; Obesity, Morbid[mh]; Body Mass Index[mh]; Bariatric Surgery[mh]; Weight Loss[mh]; obesity; obese; morbid obesity; morbidly obese; severe obesity; body mass index; BMI; bariatric; weight loss) OR (Insurance Claim Review[mh]; administrative claims; claims database; claims data; administrative data; national database; national registry; PearlDiver; Nationwide Readmissions; National Inpatient Sample; Medicare)).

S3, host and pathogen genomics in bone and joint infection: (Polymorphism, Single Nucleotide[mh]; Genetic Predisposition to Disease[mh]; High-Throughput Nucleotide Sequencing[mh]; Metagenomics[mh]; single nucleotide polymorphism; SNP; metagenomic next-generation sequencing; mNGS; next-generation sequencing; whole-genome sequencing; 16S rRNA) AND (Osteomyelitis[mh]; Prosthesis-Related Infections[mh]; Arthritis, Infectious[mh]; osteomyelitis; periprosthetic joint infection; prosthetic joint infection; PJI; septic arthritis; bone and joint infection; implant-associated infection).

S4, diagnostic accuracy and case definitions in PJI: the infection block above AND (Sensitivity and Specificity[mh]; diagnostic accuracy; diagnostic performance; diagnostic yield; case definition; diagnostic criteria; reference standard; gold standard; sonication; sonicate; synovial fluid; synovial biomarker; tissue culture; culture-negative; alpha-defensin; leukocyte esterase; calprotectin; MSIS; EBJIS; International Consensus Meeting).

S5, antibiotic duration after one-stage revision knee arthroplasty: five blocks combined with AND, namely antibiotic agents (Anti-Bacterial Agents[mh]; antibiotic; antimicrobial); duration (Drug Administration Schedule[mh]; duration; treatment duration; short course; prolonged; suppressive); procedure (one-stage; single-stage; Reoperation[mh]; revision; exchange arthroplasty; Debridement[mh]; DAIR); joint (Arthroplasty, Replacement, Knee[mh]; total knee arthroplasty; knee replacement; TKA); and infection (Prosthesis-Related Infections[mh]; periprosthetic joint infection; PJI).

S6, outcome instruments and thresholds of clinical importance: (minimal clinically important difference; minimal important change; minimal detectable change; MCID; patient acceptable symptom state; responsiveness; Patient Reported Outcome Measures[mh]; PROM) AND (Wrist[mh]; Wrist Joint[mh]; Carpal Bones[mh]; Osteonecrosis[mh]; wrist; carpal; lunate; Kienböck; DASH; PRWE; Mayo wrist).

Because neither Scopus nor Web of Science indexes with a controlled vocabulary, the six strategies were translated for those databases with the MeSH terms carried by the corresponding free-text expressions, searched as TITLE-ABS-KEY in Scopus and as a topic search in Web of Science, under the same language limit and date cap. The six strategies returned 17,004 records in MEDLINE, of which 16,353 were unique, together with 4812 records in Scopus and 3996 in Web of Science, giving 25,161 records identified across the three databases before duplicates between databases were removed.

**Figure 2:**
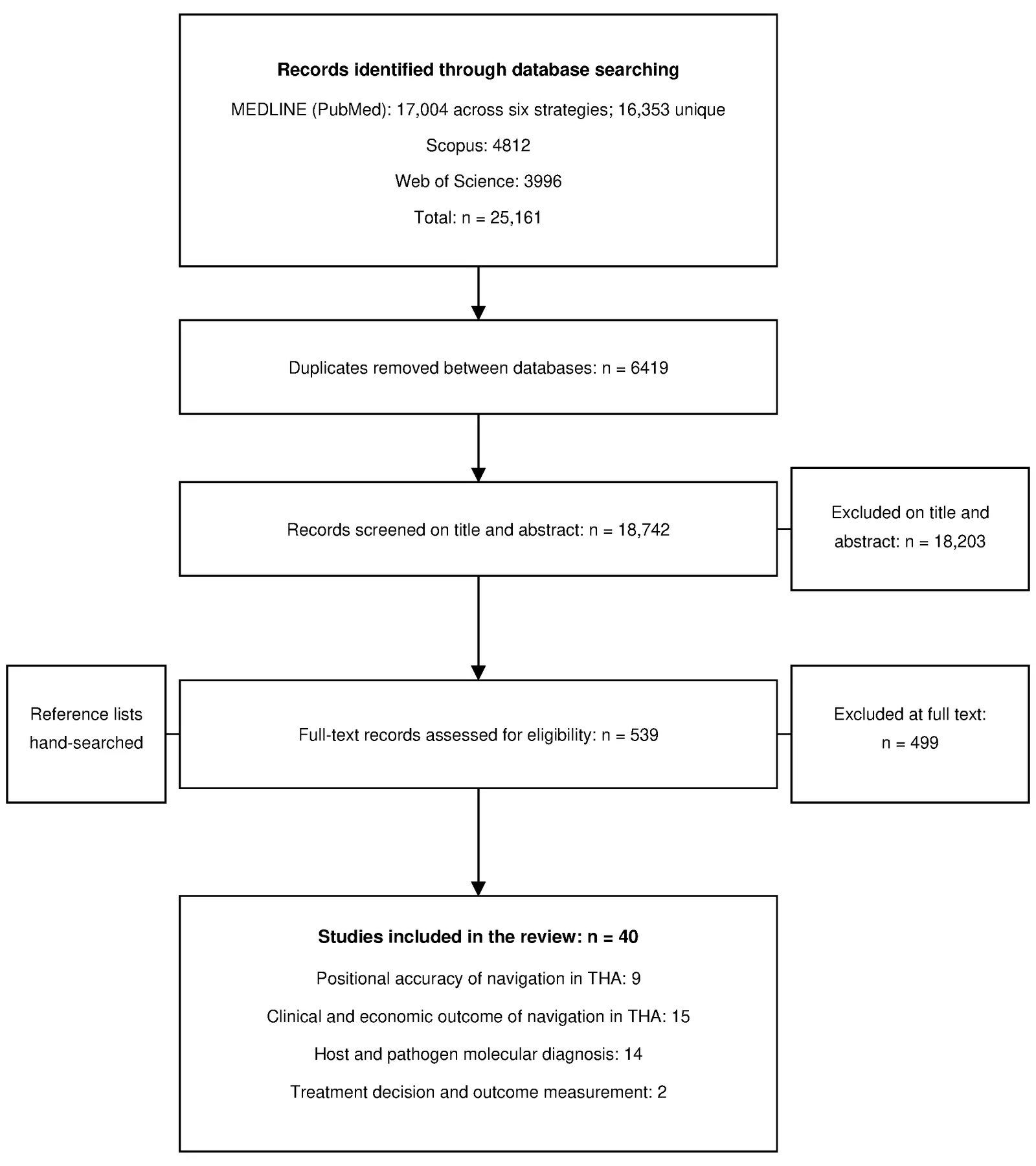
Flow of records from the documented search to the 40 studies included in this review. The MEDLINE (PubMed) figure is the yield of the six strategies defined in Section 2.2, executed with the stated filters and date cap, with records retrieved by more than one strategy counted once. The Scopus and Web of Science figures are the yields of the same six strategies translated for those databases. Full-text assessment included the records identified by hand-searching reference lists. THA, total hip arthroplasty.

S6 was run to establish whether a validated threshold of clinical importance exists for the instruments used in this field, rather than to assemble a body of studies, and its yield is reported in Figure 2 on that basis. Reference lists of retrieved reviews were hand-searched. Three included studies [23,27,28] were not retrieved by any of the six strategies and were identified in this way: one concerns robotic total knee rather than hip arthroplasty [27], and two address cup position and spinopelvic mechanics without using a navigation or robotic term [23,28]. The yield of each strategy and the flow of records to the final set of included studies are shown in Figure 2.

### 2.3. Eligibility Criteria

Inclusion criteria: Studies in human subjects, published in English, were eligible if they addressed one of the four questions defined in the Introduction and reported at least one of the following: (i) a measure of positional accuracy of component placement in THA, such as the mean absolute angular error, outlier rate, or proportion within a defined safe zone; (ii) a measure of diagnostic performance in bone or joint infection, such as sensitivity, specificity, or the detection rate against a stated case definition; (iii) a patient-level clinical outcome after THA or after treatment of PJI or osteomyelitis, such as infection, dislocation, periprosthetic fracture, mechanical loosening, revision, readmission, eradication, or a patient-reported score; (iv) a patient-level economic outcome, such as cost, cost-effectiveness, or a health technology assessment; or (v) a factor proposed to modify the relation between accuracy and outcome, such as the patient risk profile, spinopelvic mechanics, prior antibiotic exposure, or the case definition applied. Systematic reviews, meta-analyses, and umbrella reviews were eligible where they synthesised one of these questions; narrative reviews were eligible only where they are the sole available synthesis of a question, which applies to the host genomic literature [12] and to the outcome measurement example [25].

Exclusion criteria: Animal studies, in vitro and cadaveric studies, biomechanical and purely computational studies without a clinical series, conference abstracts, editorials, letters, case reports and case series of fewer than ten patients, non-English publications, and reports from which no accuracy, diagnostic performance, clinical, or economic outcome could be extracted were excluded. Studies of navigation or robotics in joints other than the hip were excluded, except where an analysis of them bears directly on the magnitude of effect that a hip navigation study would need to detect, which applies to two pooled analyses and one cohort [27,29,30]. Studies of conditions other than hip osteoarthritis treated by THA, PJI, and osteomyelitis were excluded, with the single exception described in Section 4.4.

Priority rules: Where several studies addressed the same question, preference was given in the following order: randomised trials; prospective matched or propensity-matched cohorts; systematic reviews and meta-analyses; retrospective cohorts; and, last, narrative syntheses. Within each level, studies reporting a patient-level outcome were preferred over those reporting accuracy alone, and studies published from 2021 onward were preferred. Older sources were retained only where they define a reporting framework, an appraisal tool, or a diagnostic reference standard, which applies to references [18,31,32].

### 2.4. Study Selection and Data Extraction

Records were screened by title and abstract and then in full text. Eligibility was agreed upon by discussion among the authors; screening was not duplicated independently. The flow of records from identification to inclusion is given in Figure 2. For each included study, the following were extracted into Table 1: first author, year of publication, study design, setting and sample size, and the main finding bearing on the accuracy-to-outcome question. Where a study reported an effect estimate, the estimate was extracted together with its 95% confidence interval and *p*-value as published; where the original report gives a *p*-value without an interval, or an interval without a *p*-value, that is stated in Table 1 and in the text.

### 2.5. Critical Appraisal

No formal risk-of-bias instrument was applied, and no quality score was assigned because this is a narrative review. To apply judgement consistently rather than impressionistically, each included study was nevertheless read against the same five items: study design; the reference standard or case definition used; the method of controlling confounding; whether and how multiplicity across outcomes and time points was handled; and whether confidence intervals were reported, in particular for null results. These five items are reported for the two navigation outcome cohorts in Table 2 and, for the diagnostic studies, through the reference standard column of Table 3. Where a study reports routinely collected health data, the design features relevant to the REporting of studies Conducted using the Observational Routinely collected health Data (RECORD) framework [31] were noted because those features determine what such a study can and cannot support. General guidance on the preparation and reporting of orthopaedic studies was consulted where it bears on the evidence standards discussed in Section 4.2 [33].

## 3. Results

The results are presented in nine subsections. Each subsection names the outcome it concerns, lists the studies that report that outcome, and states what those studies found. Section 3.1, Section 3.2, Section 3.3 and Section 3.4 concern THA; Section 3.5, Section 3.6 and Section 3.7 concern bone and joint infections; Section 3.8 and Section 3.9 concern the two endpoints through which any benefit of either technology would have to appear. All included studies are tabulated in Table 1.

### 3.1. Navigation in THA: Positional Accuracy (Outcome: Component Position)

Nine studies reported component position as the outcome [1,2,3,4,5,6,7,8,9]. The accuracy data are consistent. Under the conditions described below, navigation systems do what they claim to do at the level of component position. An accelerometer-based imageless system improved acetabular cup placement accuracy in the lateral decubitus position [1]. Registration workflow alters navigation error without altering the orientation finally achieved. In 46 propensity-matched pairs undergoing lateral decubitus THA, postoperative cup orientation was similar between supine and lateral registration workflows (radiographic anteversion 15.2° ± 3.3° versus 14.6° ± 5.9°, *p* = 0.56), while the mean absolute anteversion error was lower with supine registration (2.2° ± 1.5° versus 4.1° ± 3.4°, *p* < 0.001), and lateral registration tripled the odds of an error beyond 5° (odds ratio [OR] 3.11; 95% confidence interval [CI] 1.36 to 7.09; *p* = 0.007) [2]. Computed tomography (CT)-based navigation and accelerometer-based portable navigation have been compared directly in dysplastic hip osteoarthritis [3]. A validation study used a CT-based and an imageless portable system in the same procedures, and accuracy was comparable between them [4].

The accuracy is conditional rather than absolute. In imageless systems used for the direct anterior approach, the reference plane dominates the result: mean absolute anteversion error was 2.4° ± 1.8° using the table tilt plane against 9.1° ± 6.2° using the anterior pelvic plane (*p* < 0.001), and the authors conclude that a functional reference plane outperforms an anatomic one [5]. A three-arm comparison of conventional fluoroscopy, fluoroscopic image-dependent navigation, and imageless navigation in 150 patients undergoing direct anterior THA found image-dependent navigation the most accurate of the three, while imageless navigation required the longest operative time (75.5 ± 10.8 versus 65.8 ± 8.5 min for conventional fluoroscopy, *p* < 0.001) [6]. Agreement with an independent postoperative reference is weaker than agreement with the system’s own plan: imageless navigation showed limited anteversion agreement with postoperative EOS (EOS imaging, Paris, France) assessment in lateral decubitus THA [7]. Augmented reality guidance carries its own error structure, and the risk factors for that error have been identified [8]. Mean errors can be small while outlier rates are not. An augmented reality-based portable system produced mean absolute errors of 1.8° ± 1.7° for inclination and 2.0° ± 1.2° for anteversion, yet leg lengthening outliers of 5 mm or more occurred in 29.4% of supine and 6.5% of lateral decubitus cases (*p* = 0.031), and offset outliers occurred in roughly a quarter of cases in both positions [9].

Findings for this outcome: Across nine studies, navigation reduces positional outliers under defined registration conditions, and platforms are separable from one another on accuracy. The literature is internally coherent, and no study contradicts it. This outcome is a property of the implant position, not of the patient.

### 3.2. Navigation in THA: Clinical Outcome in Morbid Obesity (Outcomes: Infection, Dislocation, Periprosthetic Fracture, Mechanical Loosening, and Revision)

Two studies reported patient-level clinical outcomes after navigated THA [10,11]. Both were matched cohort studies, both drew on the same administrative claims source, and both were restricted to patients who have morbid obesity, defined as a body mass index at or above 40 kg/m^2^, undergoing elective primary THA. No study in the search reported patient-level clinical outcomes of navigated THA in an unselected population; therefore, the findings below apply to this subgroup and cannot be extended to THA patients in general. Both reports give an odds ratio with a 95% confidence interval for every comparison, and those estimates are reproduced in Table 2.

In the imageless navigation study, patients were identified in the Mariner dataset via the PearlDiver M170Ortho platform (PearlDiver Technologies, Colorado Springs, CO, USA) between 2010 and 2021, and cases performed with robotic assistance or image-based navigation were excluded [10]. Matching was 1:3 on age, sex, year of procedure, and comorbidities. After matching, 4499 patients were analysed, of whom 3367 (74.8%) were manual, and 1132 (25.2%) were imageless navigation cases. Fourteen outcomes were assessed across 35 comparisons by univariable regression. None reached significance. At two years, the odds ratios were 1.10 (95% CI 0.81 to 1.49; *p* = 0.592) for periprosthetic joint infection, 1.34 (0.86 to 2.08; *p* = 0.244) for dislocation, 1.14 (0.64 to 2.03; *p* = 0.78) for periprosthetic fracture, 1.62 (0.94 to 2.80; *p* = 0.113) for mechanical loosening, 1.23 (0.79 to 1.92; *p* = 0.426) for aseptic revision, and 0.86 (0.46 to 1.60; *p* = 0.739) for septic revision. Thromboembolic events, emergency department visits, and readmission likewise did not differ. One result deserves comment: leg length discrepancy at two years is reported as OR 1.89 (95% CI 1.02 to 3.47; *p* = 0.058). The interval excludes unity while the *p*-value does not fall below 0.05, which is internally inconsistent and presumably reflects an interval taken from the regression model and a *p*-value from a chi-square test. The authors describe this result as approaching significance and attribute it to limited power.

The image-based navigation study used the same Mariner dataset via the same PearlDiver platform, with the same population definition, and 1:3 matching on age, sex, year of surgery, diabetes, hypertension, tobacco use, and alcohol use [11]. After matching, 5835 patients were analysed, comprising 1473 navigation-assisted and 4362 manual cases. Eleven outcomes were assessed across 21 comparisons at 90 days, one year, and two years, with surgical site infection also at 30 days and transfusion on the day of surgery. At two years, the odds ratios were 0.79 (95% CI 0.56 to 1.10; *p* = 0.184) for periprosthetic joint infection, 0.70 (0.45 to 1.12; *p* = 0.163) for dislocation, 0.84 (0.44 to 1.61; *p* = 0.722) for mechanical loosening, 1.23 (0.85 to 1.80; *p* = 0.320) for aseptic revision, and 0.59 (0.25 to 1.42; *p* = 0.319) for septic revision; periprosthetic fracture at one year was 0.92 (0.52 to 1.66; *p* = 0.906). One comparison reached significance. Same-day blood transfusion occurred in 0.8% of navigation and 1.9% of manual cases, OR 0.42 (95% CI 0.23 to 0.77; *p* = 0.005). That single result sits within a family of 21 comparisons, and the report does not state whether or how multiplicity across that family was handled. It is reported here as published, without adjustment, and no mechanism linking navigation to transfusion requirement is proposed by the investigators or apparent to us.

Findings for these outcomes: Two navigation modalities were tested in two matched cohorts of patients with morbid obesity, and neither was associated with a reduction in any clinical outcome at the time points assessed. Because both reports give confidence intervals, what the two cohorts can and cannot exclude is read directly from them rather than inferred. For periprosthetic joint infection at two years, the intervals run from 0.81 to 1.49 in one cohort, and from 0.56 to 1.10 in the other; for dislocation at two years, from 0.86 to 2.08 and from 0.45 to 1.12. Intervals of this width exclude a large benefit and are equally consistent with a moderate benefit and with moderate harm. Two further qualifications are not matters of interpretation. The two cohorts share investigators, a data source, a population definition, and an overlapping selection period, so they are not independent confirmations of one another (Section 4.3). And the outcomes concerned are uncommon events assessed over two years in cohorts of a few thousand patients. For these reasons, the two cohorts are treated here as evidence against a large benefit of navigation in this subgroup, and not as evidence that navigation confers no benefit at all.

### 3.3. Navigation in THA: Factors That May Explain the Absent Clinical Signal (Outcome: Readmission, Reoperation, and Revision)

Ten studies bear on why a positional advantage might not appear as a clinical one [23,24,27,28,29,30,34,35,36,37]. Four explanations are compatible with the data, and they are not mutually exclusive.

The first is that outcome risk in this population is dominated by factors navigation does not address. Severe obesity is independently associated with higher early complications, readmission, reoperation, and revision after elective primary THA. One propensity-matched analysis drew on the Nationwide Readmissions Database for 2020 to 2022. Severe obesity was present in 7.9% of 366,374 weighted elective THAs. After matching, it was associated with 90-day readmission (OR 1.32; 95% CI 1.23 to 1.41; *p* < 0.001), hip-related reoperation (OR 1.57; 95% CI 1.39 to 1.77; *p* < 0.001), and component-level revision (OR 1.53; 95% CI 1.33 to 1.76; *p* < 0.001) [34]. If soft tissue envelope, wound healing, and metabolic state drive these events, component position is not the operative variable.

Interventions aimed directly at the metabolic state have also underperformed. A population-based cohort from Ontario included 148,977 THA patients. Prior bariatric surgery did not reduce major surgical complications. Patients who achieved a body mass index below 40 after bariatric surgery still had higher odds of major complications (OR 1.84; 95% CI 1.16 to 2.94) and of periprosthetic joint infection (OR 3.36; 95% CI 1.48 to 7.63) than patients at a similar body mass index without prior bariatric surgery [35]. The surgical approach behaves similarly. In morbidly obese patients, the direct anterior approach shortened the operative time compared with the postero-lateral approach (71 ± 18 versus 92 ± 26 min, *p* < 0.001), but adjusted complication risks were otherwise similar between approaches [36]. Glucagon-like peptide-1 receptor agonists have been associated with lower periprosthetic joint infection and readmission rates in retrospective series, though the certainty of that evidence is low and the findings are hypothesis-generating [37].

The second explanation is that the outcomes measured are the wrong ones or are measured too early. Dislocation and revision within two years are uncommon events. The base rate is low. A claims-based cohort of a few thousand patients has limited power to detect a modest relative reduction in an outcome of that frequency. Mechanical loosening attributable to component malposition may declare itself well beyond the two-year horizon.

The third is that the positional gain is real but too small to matter clinically at the population level. This is broadly consistent with the robotics literature. An umbrella review of systematic reviews and meta-analyses concluded that robotics improves reproducibility of component positioning while producing modest but measurable clinical benefits in THA [29]. A meta-analysis of 37 studies found better anteversion (OR 2.67; 95% CI 1.15 to 6.19) and inclination accuracy (OR 2.71; 95% CI 1.47 to 4.98) with robot-assisted THA, together with a lower periprosthetic fracture rate (OR 0.54; 95% CI 0.38 to 0.77) and less leg length discrepancy (mean difference −1.42 mm; 95% CI −2.19 to −0.66), at the cost of a longer operative time (mean difference 18.16 min; 95% CI 8.81 to 27.51), while the Harris Hip Score and other complication rates were comparable between groups [30]. Robotic-assisted total knee arthroplasty with one platform has likewise been associated with improved functional outcomes [27]. Technology-assisted arthroplasty therefore does show a measurable benefit in some settings. The null findings in morbid obesity should be read alongside that evidence and not as a general verdict on the technology. The comparison is indirect: robotic and navigated procedures are not equivalent interventions, and the populations differ. What the contrast establishes is only that the expected effect size is small rather than zero, which makes a null result in a claims-based study of a high-risk subgroup unsurprising.

A fourth explanation concerns the target rather than the technology. Positional accuracy is conventionally judged against the Lewinnek safe zone, and that reference has weakened. In a spinopelvic analysis of 78 THA subjects, prosthetic impingement was least frequent with an individualised cup orientation (9%) compared with six commonly selected orientations (18% to 61%), while bone impingement occurred in 33% of subjects and was not affected by cup position [23]. In that series, a third of the subjects therefore carried a form of impingement that no navigation system can control. The target also moves. Among 136 patients with preoperative spinopelvic hypermobility, 95% were no longer hypermobile one year after THA, and the mean standing-to-seated change in sacral slope fell from 36.4° (SD 5.1°) before surgery to 21.4° (SD 6.6°) at one year (*p* < 0.001)—a shift accompanied by a mean 8.5° (SD 5.5°) decrease in seated functional cup anteversion [28]. A component placed precisely against an intraoperative target may therefore sit differently in function once spinopelvic mechanics adapt. A randomised comparison of 37 hips is consistent with this: 1 of 17 robotic-arm-assisted and 8 of 20 conventional manual components fell outside the Lewinnek safe zone, and there was no dislocation in either group [24].

Findings for these outcomes: In this population, measured risk is driven by factors that navigation does not act on, the events of interest are too infrequent to be excluded at two years, the effect size suggested by the robotics literature is small, and the positional target against which accuracy is judged is itself both imperfect and unstable after surgery. Each of these would independently attenuate a clinical signal, and they act together.

### 3.4. Navigation in THA: Cost (Outcome: Cost and Cost-Effectiveness)

Three sources reported cost as the outcome [38,39,40]. If the clinical increment is small, cost becomes decisive. Economic evaluation of robotic-assisted versus manual THA has been reviewed systematically [38]. Computer-assisted fluoroscopic navigation has been compared for cost-effectiveness against robotic and manual techniques in primary THA [39]. The National Institute for Health and Care Excellence early value assessment of robotic-assisted surgery for orthopaedic procedures has been summarised for the orthopaedic readership [40].

Findings for this outcome: None of the three economic analyses was conducted in patients with morbid obesity, so none of them can be applied directly to the two cohorts in Section 3.2. The relevance is structural rather than numerical: in a population in which no clinical advantage was detected at two years, the acquisition, maintenance, and operative time costs of navigation stand unopposed by any measured benefit, and the only candidate offset identified anywhere in these data is the same-day transfusion difference of 1.1 percentage points reported in one cohort [11], which is unadjusted for multiplicity and has no proposed mechanism.

### 3.5. Host Genomics in Osteomyelitis (Outcome: Susceptibility to and Resolution of Infection)

One synthesis reported this outcome [12]. Osteomyelitis is marked by chronic inflammation, diagnostic uncertainty, and recurrence despite debridement, antimicrobial therapy, and reconstruction. Recurrence remains common. Candidate gene association work has identified immunogenetic variants that track with susceptibility and with resolution. A narrative synthesis of single nucleotide polymorphism (SNP) association studies grouped these into pro-inflammatory variants associated with increased susceptibility to chronic or post-traumatic osteomyelitis, including IL-1β rs16944 and NLRP3 rs10754558, and variants within NOS2 and VDR that are associated with protective effects that may favour infection resolution [12]. The proposed mechanisms are differences in inflammasome activity, cytokine signalling, and oxidative stress responses.

The clinical claim implied by this literature is stratification: that a patient’s genotype could identify who will recur and who will clear. That claim is not yet testable in practice. Candidate gene studies are individually small. Replication across populations is limited. No prospective cohort has compared a genotype-informed treatment decision against standard care. Large-scale validation of candidate SNPs and translational genomic models remains the prerequisite for clinical application [12].

Findings for this outcome: Associations between host genotype and the course of osteomyelitis have been reported, but no study has yet measured a patient outcome under a genotype-informed decision. This is an absence of evidence, and it is a different situation from Section 3.2, where an outcome was measured and no benefit was found.

### 3.6. Pathogen-Side Molecular Diagnosis in PJI (Outcome: Sensitivity and Specificity)

Five studies reported diagnostic performance as the outcome [13,14,15,16,17] and are collected in Table 3. Throughout this section, the index test is the diagnostic method whose performance is being measured, such as metagenomic next-generation sequencing, culture, or sonication fluid culture, and the reference standard is the independent criterion against which each case is classified as infected or not infected, such as the 2011 Musculoskeletal Infection Society (MSIS) criteria. Sensitivity and specificity are always properties of an index test relative to a particular reference standard rather than properties of the test alone.

Pathogen-side molecular diagnostics are further advanced than the host-side work and expose a different problem. Metagenomic next-generation sequencing (mNGS) has been reviewed for its role in periprosthetic joint infection diagnosis [13], its application value and challenges after arthroplasty have been assessed directly [14], and targeted next-generation sequencing (NGS) has been positioned within a proposed clinical algorithm for the detection of periprosthetic joint infection [15]. The measured performance is more modest than the enthusiasm. In a prospective study of suspected knee periprosthetic joint infection, 215 patients were enrolled and 58 entered the final analysis, of whom 38 had periprosthetic joint infection. Against the 2011 MSIS criteria, mNGS achieved a sensitivity of 63.2% (95% CI 53.6 to 77.7) and a specificity of 80.0% (95% CI 75.7 to 90.1), culture achieved 68.4% (95% CI 58.2 to 78.9) and 95.0% (95% CI 84.6 to 99.8), and histopathology achieved 52.6% (95% CI 46.1 to 68.7) and 100% (95% CI 100.0 to 100.0) [16]. mNGS did not outperform conventional methods on either axis, and the authors identify a high false-positive rate, interpretive difficulty, and the absence of susceptibility data as limits on its use. The reference standard used deserves attention. This particular study applied the 2011 MSIS criteria, and in the external validation cohort of the 2018 evidence-based definition, the 2011 MSIS criteria had a sensitivity of 79.3% against 97.7% for the new definition, at comparable specificity [18]. An index test evaluated against the older criteria is therefore calibrated against a less sensitive reference standard, under which some infections are classified as aseptic, and its accuracy estimates may not transfer to settings that apply the 2018 definition.

The case for culture-independent methods rests less on raw accuracy than on robustness to prior antibiotic exposure. Culture positivity fell from 86.3% in patients off antibiotics to 54.8% after more than seven days of exposure, while mNGS fell from 92.2% to 76.2%, and mNGS detected more pathogens than culture in both the short-term (86.7% versus 70.0%, *p* = 0.027) and long-term exposure groups (76.2% versus 54.8%, *p* = 0.039) [17].

Findings for this outcome: Against a defined case standard, mNGS is not more accurate than culture, and is less specific. Its measurable advantage is confined to the patient who has already received antibiotics, in whom culture loses roughly a third of its yield. This is a real but narrow indication, and it is an advantage in detection rather than in any patient outcome. All of these methods are validated against culture or against a composite case definition, which is the constraint examined next.

### 3.7. The Reference Standard in PJI (Outcome: Dependence of Measured Rates and Accuracy on the Case Definition)

Eight studies bear on this outcome [18,19,20,21,41,42,43,44]. Both the host and pathogen studies are validated against a reference standard that is unstable. The search for a gold standard in periprosthetic joint infection diagnosis remains unresolved [19]. The consequence is quantitative rather than philosophical. Rates of periprosthetic joint infection in revision hip and knee arthroplasty differ according to the definition applied [20]. A test evaluated against one definition therefore carries sensitivity and specificity estimates that are not transferable to a centre using another. Table 3 collects the performance estimates discussed in this section together with the reference standard against which each was generated.

Sonication fluid culture demonstrates this definitional dependency within a single set of specimens. Among 429 explantations for suspected periprosthetic joint infection or aseptic failure, sonication would not have altered patient management in 61 of 95 cases defined by Infectious Diseases Society of America criteria (64.2%), and detected additional virulent organisms capable of influencing antibiotic selection in eight cases (8.4%). Under European Bone and Joint Infection Society criteria, which, unlike the Infectious Diseases Society of America criteria, admit sonication into the definition, 113 cases were classified as confirmed, and 50 of those (44.2%) met the definition solely on a positive sonication culture above 50 colony-forming units per millilitre [21]. Nearly half of the confirmed cases under one definition would not have been confirmed on that basis under the other. The authors conclude that sonication influences clinical management in approximately 8% to 20% of cases. This study is purely descriptive and reports no confidence intervals or *p*-values, which is appropriate to its design and is not an omission. It is incorporation bias in textbook form: the index test constitutes part of the reference standard against which it is assessed, a risk covered explicitly by the reference standard domain of QUADAS-2 [32], and it inflates apparent agreement in a way no larger sample corrects.

This propagates. Serum and synovial biomarker thresholds for diagnosing periprosthetic joint infection have been assessed in a QUADAS-C-guided systematic review and meta-analysis [41], and the diagnostic accuracy of synovial leukocyte esterase, glucose, and calprotectin has been quantified [42]. Every such threshold is conditional on the case definition used to classify the reference cases. Cost-effectiveness analysis of hip periprosthetic joint infection diagnostic pathways has been performed using the European Bone and Joint Infection Society and MSIS definitions [43], which makes the definitional dependency explicit rather than implicit. The organism identified also carries prognostic weight independent of the test that identified it: Gram-negative periprosthetic joint infection is followed by a high and early failure rate in a retrospective cohort of 72 cases [44].

Findings for this outcome: The quantity against which every diagnostic method in this field is measured is itself a choice. Changing the definition changes the measured infection rate, changes the apparent sensitivity of the test, and in the case of sonication, determines whether nearly half of the confirmed cases are confirmed at all. No improvement in analytical sensitivity can correct this because it is a property of the comparator and not of the test.

### 3.8. The Treatment Decision That Diagnosis Is Meant to Inform (Outcome: Infection Eradication)

One systematic review reported eradication as the outcome [22]. A diagnostic or genomic test can improve a patient outcome only by changing a treatment decision; therefore, the chain described in Section 3.5, Section 3.6 and Section 3.7 can only be completed where such a decision is well defined and its outcome has been measured. The decision that pathogen identification, and prospectively host genotyping, are proposed to inform is the choice and duration of antibiotic therapy after surgical treatment of infection (Section 3.5). The only synthesis of that decision against a hard outcome identified by the search concerns the knee rather than the hip and is included on that basis. The joint differs; the diagnostic question does not, since the organism is identified by the same methods and the case is defined by the same criteria in both joints.

A systematic review searched MEDLINE, Scopus, Web of Science, and the Cochrane Central Register of Controlled Trials without date, language, or design restriction. It identified 963 studies, of which 21 were included [22]. Regimens were classified as short-term intravenous therapy (two weeks or less), short-term intravenous therapy plus long-term oral therapy, and long-term intravenous therapy (minimum six weeks). Coagulase-negative staphylococcal species were the most frequently isolated pathogens. The mean eradication rate across all included studies was 88.4% (range 62.5 to 100%). Across all 21 studies, short-term intravenous plus long-term oral therapy showed a higher eradication rate than the other two regimens taken together (*p* = 0.023). Among the ten studies with more than five years of follow-up, this particular difference was no longer statistically significant. In the subgroup in which antibiotic-loaded cement was used, eradication was higher with short-term intravenous plus long-term oral therapy (92.8%) and with long-term intravenous therapy (89.7%) than with short-term intravenous therapy alone (*p* = 0.006). The review reports these comparisons as percentages with *p*-values and does not report confidence intervals for them.

Findings for this outcome: Two features bear on the argument. First, the apparent advantage of one regimen attenuated with longer follow-up, which is the same time-horizon caution that applies to the two-year navigation cohorts in Section 3.2: a difference measured early is not necessarily a difference that persists. Second, and decisively for this review, none of the 21 included studies stratified antibiotic duration by host genotype or by a molecular diagnosis. The treatment decision that host genomics is proposed to inform is, at present, made without it. The diagnostic literature of Section 3.5, Section 3.6 and Section 3.7 and the treatment literature of this section do not yet connect. Prospective and randomised studies are required to establish a patient-based protocol for the type and duration of antibiotic therapy after one-stage treatment of a knee periprosthetic joint infection [22].

### 3.9. The Instruments Through Which Benefit Would Be Detected (Outcome: Measured Function)

A benefit that cannot be measured cannot be demonstrated. Where the instrument used to detect a patient-level benefit has no validated threshold of clinical importance, the chain from measurement to outcome has no terminal link, and this applies to both settings reviewed here.

In THA, the reported endpoint of the accuracy literature is positional deviation in degrees (Section 3.1). A threshold does exist for it in the form of the Lewinnek safe zone, but that threshold is not anchored to anything the patient experiences. Components placed inside it still dislocate; an individualised orientation outperforms any fixed target for prosthetic impingement; and bone impingement, which occurred in a third of subjects in one spinopelvic series, is independent of cup position altogether [23,24]. A surrogate endpoint whose threshold does not track the patient-level event is not a stronger endpoint than one with no threshold at all.

In periprosthetic joint infection, the terminal endpoint is eradication, which is definable, but it is defined by the same case criteria whose instability is the subject of Section 3.7. Whether a patient is counted as eradicated depends on which definition is applied; therefore, the endpoint inherits the problem rather than escaping it.

Findings for this outcome: In neither setting is there an endpoint that is both measurable and anchored to what the patient notices. In THA, the available threshold is not patient-anchored; in periprosthetic joint infection, the endpoint is definition-dependent. This is the terminal constraint on the whole argument, and it is examined further in Section 4.4, where the same gap is shown to arise in an unrelated orthopaedic condition and is therefore not specific to the hip or to infection.

## 4. Discussion

### 4.1. Principal Findings

Four findings follow from Section 3. First, navigation in THA achieves what it claims at the level of component position, consistently and across platforms (Section 3.1). Second, in the only population in which its clinical outcomes have been tested, patients with morbid obesity, that positional advantage was not accompanied by a reduction in infection, dislocation, periprosthetic fracture, mechanical loosening, or revision within two years (Section 3.2), and at least four non-exclusive mechanisms would independently attenuate such a signal (Section 3.3). Third, in bone and joint infections, the position is different in kind: host genomic stratification has never been tested against a patient outcome (Section 3.5), and pathogen-side sequencing is no more accurate than culture, except after antibiotic exposure (Section 3.6), while both are measured against a case definition that determines the result (Section 3.7). Fourth, the treatment decision that molecular diagnosis is meant to inform is currently made without it (Section 3.8), and neither setting possesses an endpoint that is both measurable and anchored to patient experience (Section 3.9).

The distinction drawn in the Introduction is therefore sustained by the evidence. For navigation in THA, the relevant statement is that a large benefit has been looked for in one high-risk population and not found. For host genomics and molecular diagnoses, it is that benefit has not been looked for at all. These are not the same conclusions, and they do not call for the same response.

### 4.2. Limits of the Current Evidence and What the Next Generation of Studies Requires

The limits identified in Section 3 are not limits of the technologies; they are limits of the studies through which the technologies have been evaluated, and three of them are remediable by design.

Outcome-anchored endpoints: Accuracy endpoints should be treated as intermediate. A navigation study should report revision, dislocation, and function; a diagnostic study should report antimicrobial change and eradication rather than concordance with culture alone, and where an intermediate endpoint is used, the study should state what evidence links that endpoint to the patient-level outcome it is standing in for. Section 3.9 is the reason this cannot be deferred: an endpoint that is not anchored to patient experience will not detect a benefit, even where one exists.

Prespecified and reported case definitions: Because periprosthetic joint infection rates vary with the applied definition [20], any study reporting infection as an endpoint should name the definition used and, where feasible, report the endpoint under more than one definition. Claims-based studies identify infection through diagnostic and procedural codes rather than the European Bone and Joint Infection Society or MSIS criteria, and this should be stated as a limitation rather than left implicit. Both cohorts in Section 3.2 are claims-based and both report infection as an outcome, so this applies to them directly.

Reporting appropriate to the data source: Studies using routinely collected health data have specific vulnerabilities: code-based exposure and outcome definitions, absent clinical granularity, and confounding by indication. The RECORD statement, an extension of the Strengthening the Reporting of Observational Studies in Epidemiology (STROBE) statement, comprising a 13-item checklist for studies using routinely collected health data, exists for this purpose [31]. Reporting the matching variables, the code lists, and the follow-up completeness allows a reader to judge whether a negative result reflects absence of an effect or absence of power. In both matched cohorts discussed here, matching variables, cohort sizes, and effect estimates with confidence intervals are reported [10,11], which is what allows the reader to see that these cohorts exclude a large effect and not a moderate one (Section 3.2). Reporting a null finding as an interval rather than as a *p*-value above 0.05 is the single change that most improves such a study because a statement that an outcome did not differ at *p* > 0.05 does not separate absence of an effect from absence of power, and where equivalence is the intended claim, a prespecified equivalence margin should be added. Where many outcomes are tested at several time points, the analysis should state how multiplicity was handled so that an isolated significant result such as the transfusion difference in Section 3.2 can be interpreted by the reader rather than adjusted after the fact by others.

To these three, a fourth applies before submission. Practical guidance on preparing and reporting orthopaedic studies for publication has been set out for this readership [33]. It applies with particular force to database studies, where analytical convenience makes it easy to produce a statistically clean result that answers no clinical question.

### 4.3. Limitations of This Review

This is a narrative review and is subject to selection bias in the sources examined. Although the search strategy, eligibility criteria, and selection process are documented in Section 2, and the included studies are listed in full in Table 1, screening was not duplicated across the whole yield, no formal risk-of-bias instrument was applied, no quality score was assigned, and no quantitative synthesis was performed. No new statistical calculation of any kind was undertaken; every estimate reported above is as published by the original investigators. Readers requiring pooled estimates or a graded certainty assessment should not look for them here.

The navigation evidence discussed in detail is drawn from two matched cohorts in a single high-risk population. The findings should not be extended to unselected arthroplasty patients, in whom the balance of risk factors differs. Both cohorts were drawn from the same source, the Mariner dataset, accessed through the PearlDiver platform [10,11], over selection periods that overlap, and they share investigators and a population definition. The degree of patient overlap between them cannot be determined from the published reports, and they should not be read as independent replications. Follow-up in both is limited to two years, which is insufficient for aseptic loosening. Navigation is not randomly allocated in either, and its adoption tracks surgeon, hospital, and case-complexity characteristics that administrative data do not capture; therefore, confounding by indication cannot be excluded in either direction. Morbid obesity is identified by diagnostic code rather than by measured body mass index, and obesity coding in administrative data is incomplete. As a result, misclassification of the study population is possible.

The genomic literature reviewed is predominantly candidate gene work rather than genome-wide, and the mNGS literature includes single-centre series alongside prospective comparisons. Several of the diagnostic estimates quoted derive from small samples: the prospective comparison in Section 3.6 analysed 58 patients, and its confidence intervals are correspondingly wide and overlapping. The review also concentrates on navigation in THA and does not systematically cover the wider robotic and navigated arthroplasty literature, including registry analyses and randomised trials in total knee arthroplasty, some of which report lower revision rates or better outcomes with technology assistance; that literature is represented here mainly through pooled analyses [27,29,30]. Finally, four of the included studies have an author in common with one another, and the search was restricted to English-language publications; therefore, relevant work in other languages was not retrieved.

### 4.4. Future Research Directions

Three studies would change the state of this field, and each is feasible now.

For navigation in THA, a registry-based or multicentre cohort in an unselected population, with revision and dislocation used as primary endpoints, along with follow-up beyond five years, a prespecified analysis plan with a stated approach to multiplicity, and effect estimates with confidence intervals reported for null as well as positive results. The existing evidence excludes a large benefit in morbid obesity; it says nothing about a small benefit, and nothing about the unselected patient.

For host genomics in osteomyelitis, a prospective cohort in which genotype is determined at presentation and recurrence is the endpoint, followed, if that succeeds, by a randomised comparison of a genotype-informed strategy against a standard antibiotic strategy. Until the first of these exists, no clinical claim for stratification can be assessed, and the candidate variants of Section 3.5 remain associations.

For molecular diagnosis in periprosthetic joint infection, a trial in which the randomised unit is the diagnostic pathway rather than the test, with antimicrobial change and eradication used as endpoints, each case classified under both the 2018 and the European Bone and Joint Infection Society definitions so that the dependence demonstrated in Section 3.7 is measured rather than assumed. The population already identified as most likely to benefit from this is the one in which culture fails, namely the patient who has received antibiotics before sampling (Section 3.6).

Underlying all three of these is the measurement problem seen in Section 3.9, and it is worth establishing whether this problem is peculiar to the hip and to infection or general to orthopaedics. One condition outside both settings suggests the latter. A narrative review of rehabilitation in Kienböck disease mapped conservative and postoperative pathways to disease stage and to the surgical procedure, covering immobilisation and orthotic management, dart-throwing-motion-plane mobilisation, sensorimotor retraining, oedema and pain control, joint-levelling osteotomy, vascularised bone grafting, limited intercarpal arthrodeses, proximal row carpectomy, wrist denervation, and salvage procedures, together with the patient-reported, performance-based, and occupational instruments used to assess them. Its authors state explicitly that no Kienböck-validated minimal clinically important difference exists, and identify the absence of condition-specific functional movement screens and of sustained return-to-work data as priority gaps [25]. The same terminal link is missing in a different bone, a different joint, and a different disease process. Without a validated minimal clinically important difference, a trial cannot be powered on a clinically meaningful threshold, and a statistically significant between-group difference cannot be interpreted as a difference patients would notice. Developing and validating such thresholds is therefore a prerequisite for, and not a refinement of, the outcome studies proposed above.

## 5. Conclusions

Navigation in total hip arthroplasty places the acetabular component accurately. In the only population in which its clinical outcomes have been tested—patients with morbid obesity—this accuracy was not associated with fewer complications or fewer revisions within two years, across two modalities, and in two matched cohorts. This excludes a large benefit in this particular subgroup and does not address either a small benefit or the unselected patient.

Molecular diagnosis of periprosthetic joint infection and host immunogenetic profiling in osteomyelitis stand differently: their benefit has not been tested rather than tested and not found, and both are measured against a case definition that determines the measurement.

In both settings, the limiting factor is the design of the studies that connect a measurement to a patient outcome rather than the precision of the measurement. Studies that name their case definition, report according to a framework appropriate to their data source, carry a patient-level endpoint as primary, and present null results with effect estimates and confidence intervals would resolve questions that further accuracy work alone cannot.

## Figures and Tables

**Table 1:** Studies included in this review, by domain and by the outcome each addresses. Methodological and reporting references [26,31,32,33] are cited in the text but are not evidence sources and are not listed here.

First Author (Year) [Ref]	Study Design	Setting and Sample	Main Finding Relevant to This Review
**Positional accuracy of navigation in THA (outcome: component position)**			
Ohyama (2024) [1]	Prospective comparative cohort	Primary THA in the lateral decubitus position; accelerometer-based imageless navigation	Imageless accelerometer-based navigation improved acetabular cup placement accuracy.
Matsumoto (2026) [2]	Propensity-matched comparative cohort, 46 pairs	Lateral decubitus THA with a portable navigation system; supine versus lateral registration	Final cup orientation similar (radiographic anteversion 15.2° ± 3.3° versus 14.6° ± 5.9°, *p* = 0.56); mean absolute anteversion error lower with supine registration (2.2° ± 1.5° versus 4.1° ± 3.4°, *p* < 0.001); lateral registration OR 3.11 (95% CI 1.36–7.09; *p* = 0.007) for error beyond 5°.
Tanaka (2025) [3]	Retrospective comparative cohort	THA for dysplastic hip osteoarthritis; CT-based versus accelerometer-based portable navigation	Direct comparison of the two platforms on component placement.
Miura (2026) [4]	Prospective validation study	CT-based and imageless portable navigation applied within the same procedures	Accuracy comparable between the two systems.
Farey (2024) [5]	Comparative accuracy study	Direct anterior THA; imageless navigation with alternative registration planes	Mean absolute anteversion error 2.4° ± 1.8° with the table tilt plane versus 9.1° ± 6.2° with the anterior pelvic plane (*p* < 0.001); a functional reference plane outperformed an anatomic one.
Braun (2026) [6]	Three-arm comparative cohort, 150 patients	Direct anterior THA; conventional fluoroscopy versus fluoroscopic image-dependent navigation versus imageless navigation	Image-dependent navigation most accurate; imageless navigation required the longest operative time (75.5 ± 10.8 versus 65.8 ± 8.5 min, *p* < 0.001).
Bauer L (2026) [7]	Retrospective comparative study	Lateral decubitus THA; imageless navigation versus postoperative EOS assessment	Limited anteversion agreement with an independent postoperative reference.
Ishida (2026) [8]	Retrospective cohort	Augmented reality-guided cup placement in THA	Magnitude of, and risk factors for, augmented reality guidance error identified.
Ozaki (2024) [9]	Validation study in supine and lateral decubitus positions	Augmented reality-based portable navigation in THA	Mean absolute error 1.8° ± 1.7° for inclination and 2.0° ± 1.2° for anteversion; leg lengthening outliers of 5 mm or more in 29.4% of supine versus 6.5% of lateral decubitus cases (*p* = 0.031).
**Clinical and economic outcomes of navigation in THA (outcomes: complications, revision, cost)**			
Latifi (2026) [10]	Matched cohort study (1:3), administrative claims (PearlDiver Mariner dataset), 2010–2021	4499 patients with morbid obesity undergoing elective primary THA; imageless navigation (*n* = 1132) versus manual (*n* = 3367)	No significant difference in any of 14 outcomes across 35 comparisons within two years. At two years, odds ratios were 1.10 (95% CI 0.81–1.49) for periprosthetic joint infection, 1.34 (0.86–2.08) for dislocation, 1.14 (0.64–2.03) for periprosthetic fracture, 1.62 (0.94–2.80) for mechanical loosening, 1.23 (0.79–1.92) for aseptic revision and 0.86 (0.46–1.60) for septic revision.
Latifi (2026) [11]	Matched cohort study (1:3), administrative claims (PearlDiver Mariner dataset), 2010–2023	5835 patients with morbid obesity undergoing elective primary THA; image-based navigation (*n* = 1473) versus manual (*n* = 4362)	No significant difference in any of the 11 outcomes across 21 comparisons within two years. At two years, odds ratios were 0.79 (95% CI 0.56–1.10) for periprosthetic joint infection, 0.70 (0.45–1.12) for dislocation, 0.84 (0.44–1.61) for mechanical loosening, 1.23 (0.85–1.80) for aseptic revision and 0.59 (0.25–1.42) for septic revision. Same-day transfusion was lower with navigation, OR 0.42 (0.23–0.77), *p* = 0.005.
Maman (2026) [34]	Propensity-matched national database study (Nationwide Readmissions Database, 2020–2022)	366,374 weighted elective primary THAs; severe obesity present in 7.9%	Severe obesity associated with 90-day readmission (OR 1.32; 95% CI 1.23–1.41), hip-related reoperation (OR 1.57) and component-level revision (OR 1.53).
Lex (2026) [35]	Population-based cohort study (Ontario)	148,977 patients undergoing THA, with and without prior bariatric surgery	Prior bariatric surgery did not reduce major surgical complications; patients reaching body mass index below 40 after bariatric surgery still had higher odds of major complications (OR 1.84; 95% CI 1.16–2.94) and of PJI (OR 3.36; 95% CI 1.48–7.63).
Bauer JA (2026) [36]	Retrospective comparative cohort	Morbidly obese patients; direct anterior versus postero-lateral THA	Shorter operative time with the direct anterior approach (71 ± 18 versus 92 ± 26 min, *p* < 0.001); adjusted complication risks otherwise similar.
Tabor (2026) [37]	Systematic review	Glucagon-like peptide-1 receptor agonist use in patients undergoing arthroplasty	Lower PJI and readmission rates reported in retrospective series; certainty of evidence low and findings hypothesis-generating.
Vaishya (2026) [29]	Umbrella review of systematic reviews and meta-analyses	Robotic THA	Improved reproducibility of component positioning with modest but measurable clinical benefit.
Yang (2026) [30]	Systematic review and meta-analysis of 37 studies	Robot-assisted versus manual THA	Better anteversion (OR 2.67; 95% CI 1.15–6.19) and inclination accuracy (OR 2.71; 95% CI 1.47–4.98); lower periprosthetic fracture rate (OR 0.54; 95% CI 0.38–0.77); less leg length discrepancy (mean difference −1.42 mm; 95% CI −2.19 to −0.66); longer operative time (mean difference 18.16 min; 95% CI 8.81–27.51); Harris Hip Score comparable.
Sodhi (2025) [27]	Retrospective comparative cohort	Robotic-assisted total knee arthroplasty with one platform	Improved functional outcomes.
Dennis (2023) [23]	Computer simulation applied to a prospective spinopelvic series of 78 THA subjects	Cup orientation and impingement after THA	Prosthetic impingement was least frequent with individualised cup orientation (9%) versus six commonly selected orientations (18–61%); bone impingement occurred in 33% of subjects and was not affected by cup position.
Sculco (2021) [28]	Prospective cohort, 136 patients	Preoperative spinopelvic hypermobility before and one year after THA	95% were no longer hypermobile at one year; the mean standing-to-seated change in sacral slope fell from 36.4° (SD 5.1°) before surgery to 21.4° (SD 6.6°) at one year (*p* < 0.001), accompanied by a mean 8.5° (SD 5.5°) decrease in seated functional cup anteversion.
Giertych (2024) [24]	Randomised comparison, 37 hips	Robotic-arm-assisted versus conventional manual THA	1 of 17 robotic and 8 of 20 manual components fell outside the Lewinnek safe zone; no dislocation occurred in either group.
Wignadasan (2026) [38]	Systematic review of economic evaluations	Robotic-assisted versus manual THA	Synthesis of cost and clinical outcome data.
Bernstein (2026) [39]	Cost-effectiveness analysis	Computer-assisted fluoroscopic navigation versus robotic and manual primary THA	Comparative cost-effectiveness of the three techniques.
Clement (2026) [40]	Summary of a health technology assessment	NICE early value assessment of robotic-assisted surgery for orthopaedic procedures	Assessment summarised for the orthopaedic readership.
**Host and pathogen molecular diagnoses in bone and joint infection (outcomes: susceptibility, sensitivity, and specificity)**			
Hoveidaei (2026) [12]	Narrative review of candidate gene association studies	Chronic and post-traumatic osteomyelitis	Pro-inflammatory variants (IL-1β rs16944, NLRP3 rs10754558) associated with increased susceptibility; NOS2 and VDR variants associated with protective effects. Large-scale validation remains the prerequisite for clinical use.
Fida and Tande (2024) [13]	Narrative review	Metagenomic sequencing in PJI	Role and current limitations of metagenomic sequencing described.
Huang (2025) [14]	Narrative review	mNGS for PJI after arthroplasty	Application value and challenges assessed directly.
Uribarri (2026) [15]	Diagnostic study with proposed algorithm	Targeted next-generation sequencing for PJI detection	Targeted NGS positioned within a proposed clinical algorithm.
Wei (2025) [16]	Prospective diagnostic accuracy study; 215 enrolled, 58 analysed, 38 infected	Suspected knee PJI; periprosthetic tissue samples	Against the 2011 MSIS criteria, sensitivity and specificity were 63.2% and 80.0% for mNGS, 68.4% and 95.0% for culture, and 52.6% and 100% for histopathology. mNGS did not outperform conventional methods on either axis.
Parvizi (2018) [18]	Derivation and external validation of a case definition	Hip and knee PJI	In the external validation cohort, the 2011 MSIS criteria had a sensitivity of 79.3% against 97.7% for the 2018 evidence-based definition, at comparable specificity.
Lin (2025) [17]	Retrospective comparative diagnostic study	PJI with and without prior antibiotic exposure	Culture positivity fell from 86.3% off antibiotics to 54.8% after more than seven days of exposure, while mNGS fell from 92.2% to 76.2%; mNGS detected more pathogens than culture with short-term (86.7% versus 70.0%, *p* = 0.027) and long-term exposure (76.2% versus 54.8%, *p* = 0.039).
Sandu (2025) [19]	Narrative review	PJI diagnosis	No gold standard has been established.
Streck (2025) [20]	Retrospective cohort	Revision hip and knee arthroplasty	Measured PJI rates differ significantly according to the definition applied.
Zouitni (2025) [21]	Retrospective microbiology-driven cohort, 429 explantations	Suspected PJI or aseptic failure	Sonication would not have altered management in 61 of 95 IDSA-defined cases (64.2%) and detected an additional virulent organism in 8 cases (8.4%); under EBJIS criteria, 50 of 113 confirmed cases (44.2%) met the definition solely on a positive sonication culture above 50 CFU/mL.
Sartori (2026) [41]	Systematic review and meta-analysis, QUADAS-C guided	Serum and synovial biomarker thresholds for PJI	Threshold accuracy is conditional on the case definition used to classify the reference cases.
Alkadhem (2026) [42]	Diagnostic accuracy study	Synovial leukocyte esterase, glucose, and calprotectin for PJI	Accuracy of each marker quantified.
Diaz-Ledezma (2026) [43]	Cost-effectiveness analysis	Hip PJI diagnostic pathways under the EBJIS/MSIS definition	Dependence of pathway cost-effectiveness on the applied definition made explicit.
Schlossmacher (2026) [44]	Retrospective cohort, 72 cases	Gram-negative PJI	High and strikingly early failure rate.
**Treatment decision and outcome measurement (outcomes: eradication, measured function)**			
Hoveidaei (2025) [22]	Systematic review; 963 records screened, 21 studies included	Duration of systemic antibiotic therapy after one-stage revision total knee arthroplasty for PJI	Mean eradication rate 88.4% (range 62.5–100%). Short-term intravenous plus long-term oral therapy gave a higher eradication rate than the other regimens (*p* = 0.023), a difference no longer significant among the ten studies with more than five years of follow-up. With antibiotic-loaded cement, eradication was higher with short-term intravenous plus long-term oral (92.8%) and long-term intravenous therapy (89.7%) than with short-term intravenous therapy alone (*p* = 0.006). No included study stratified antibiotic duration by host genotype or molecular diagnosis.
Tenev (2026) [25]	Narrative review	Rehabilitation in Kienböck disease	No Kienböck-validated minimal clinically important difference exists; condition-specific functional movement screens and sustained return-to-work data are absent.

CFU, colony-forming units; CI, confidence interval; CT, computed tomography; EBJIS, European Bone and Joint Infection Society; IDSA, Infectious Diseases Society of America; mNGS, metagenomic next-generation sequencing; MSIS, Musculoskeletal Infection Society; NGS, next-generation sequencing; OR, odds ratio; PJI, periprosthetic joint infection; SD, standard deviation; THA, total hip arthroplasty.

**Table 2:** Design, findings, and appraisal of the two matched claims-based cohort studies of navigation-assisted total hip arthroplasty in patients with morbid obesity (body mass index ≥ 40 kg/m^2^) [10,11], reported against the five appraisal items defined in Section 2.5. Odds ratios are given as published, with their 95% confidence intervals and *p*-values.

Appraisal Item	Imageless Navigation [10]	Image-Based Navigation [11]
Study design	Matched cohort study, 1:3 matching	Matched cohort study, 1:3 matching
Data source; period	Mariner dataset via the PearlDiver M170Ortho platform; 2010–2021	Mariner dataset via the PearlDiver M170Ortho platform; 2010–2023
Matching variables	Age, sex, year of procedure, comorbidities	Age, sex, year of surgery, diabetes, hypertension, tobacco use, alcohol use
Patients analysed	4499 (1132 navigation; 3367 manual)	5835 (1473 navigation; 4362 manual)
Follow-up	30 and 90 days, 1 year, 2 years, by outcome	90 days, 1 year, 2 years, by outcome; surgical site infection also at 30 days
Outcomes and comparisons assessed	14 outcomes, 35 comparisons	11 outcomes, 21 comparisons
Periprosthetic joint infection, 2 years	OR 1.10 (95% CI 0.81–1.49), *p* = 0.592	OR 0.79 (95% CI 0.56–1.10), *p* = 0.184
Dislocation, 2 years	OR 1.34 (0.86–2.08), *p* = 0.244	OR 0.70 (0.45–1.12), *p* = 0.163
Periprosthetic fracture	OR 1.14 (0.64–2.03), *p* = 0.78 at 2 years	OR 0.92 (0.52–1.66), *p* = 0.906 at 1 year
Mechanical loosening, 2 years	OR 1.62 (0.94–2.80), *p* = 0.113	OR 0.84 (0.44–1.61), *p* = 0.722
Aseptic revision, 2 years	OR 1.23 (0.79–1.92), *p* = 0.426	OR 1.23 (0.85–1.80), *p* = 0.320
Septic revision, 2 years	OR 0.86 (0.46–1.60), *p* = 0.739	OR 0.59 (0.25–1.42), *p* = 0.319
Isolated significant difference	None. Leg length discrepancy at 2 years is reported as OR 1.89 (1.02–3.47), *p* = 0.058, in which the interval excludes unity although the *p*-value does not	Same-day transfusion, 0.8% versus 1.9%, OR 0.42 (0.23–0.77), *p* = 0.005
Statistical approach	Univariable regression; odds ratios with 95% confidence intervals reported for every comparison	Univariable regression; odds ratios with 95% confidence intervals reported for every comparison
Multiplicity across outcomes and time points	Not stated	Not stated
Control of confounding	Matching only; no multivariable adjustment reported	Matching only; no multivariable adjustment reported
Main design limitations	Claims data; confounding by indication; morbid obesity identified by diagnostic code; same data source and overlapping selection period as the image-based cohort, so patient overlap is likely but cannot be quantified	Claims data; confounding by indication; morbid obesity identified by diagnostic code; the study period is given as 2010–2023 in the abstract, and as January 2010 to March 2021 in the Methods; same data source as the imageless cohort, so patient overlap is likely but cannot be quantified
Take-home message	Imageless navigation was not associated with a lower rate of any complication or of revision within two years in patients with morbid obesity. The confidence intervals exclude a large benefit and remain compatible with a moderate effect in either direction	Image-based navigation was not associated with a lower rate of any complication or of revision within two years in patients with morbid obesity. The single transfusion difference is unadjusted for multiplicity and has no proposed mechanism

CI, confidence interval; OR, odds ratio. An odds ratio above 1 favours the manual arm, and an odds ratio below 1 favours the navigation arm.

**Table 3:** Reported performance of diagnostic methods for periprosthetic joint infection, the reference standard on which each estimate depends, and the finding each study contributes.

Setting and Design	Index Test	Reported Performance	Reference Standard	Take-Home Message
Prospective study of suspected knee PJI; 58 analysed, of whom 38 were infected [16]	mNGS; culture; histopathology	Sensitivity (95% CI)/specificity (95% CI): mNGS 63.2% (53.6–77.7)/80.0% (75.7–90.1); culture 68.4% (58.2–78.9)/95.0% (84.6–99.8); histopathology 52.6% (46.1–68.7)/100% (100.0–100.0)	2011 MSIS criteria	mNGS is neither more sensitive nor more specific than culture. The confidence intervals overlap throughout, and the sample is small.
Validation of PJI case definitions [18]	2011 MSIS criteria assessed as the index test	Sensitivity 79.3%, versus 97.7% for the 2018 definition, at comparable specificity	External validation cohort (PJI with later reinfection versus aseptic revision)	The reference standard used in [16] is itself about 18 percentage points less sensitive than the current one, so accuracy measured against it does not transfer.
PJI with prior antibiotic exposure [17]	mNGS versus culture	Positivity off antibiotics: mNGS 92.2%, culture 86.3%; after more than 7 days of exposure: 76.2% versus 54.8%; mNGS higher with short-term (86.7% versus 70.0%, *p* = 0.027) and long-term exposure (76.2% versus 54.8%, *p* = 0.039)	Head-to-head detection rates within the same patients	The one setting in which mNGS clearly outperforms culture is the patient already treated with antibiotics. This is an advantage in detection, not in outcome.
429 explantations for suspected PJI or aseptic failure [21]	Sonication fluid culture	Management unchanged in 64.2% of IDSA-defined cases; additional virulent organism in 8.4%; 44.2% of EBJIS-confirmed cases confirmed solely on sonication culture above 50 CFU/mL (descriptive study; no CIs or *p*-values reported)	IDSA versus EBJIS criteria; the index test forms part of the EBJIS definition (incorporation bias)	In the same set of specimens, sonication altered management in about one case in ten under the IDSA definition, while under the EBJIS definition it supplied the sole basis for 44.2% of confirmed diagnoses.

The index test is the diagnostic method under evaluation; the reference standard is the independent criterion used to classify each case as infected or not infected (Section 3.6). CFU, colony-forming units; CI, confidence interval; EBJIS, European Bone and Joint Infection Society; IDSA, Infectious Diseases Society of America; mNGS, metagenomic next-generation sequencing; MSIS, Musculoskeletal Infection Society; PJI, periprosthetic joint infection.

## Data Availability

No new data were created or analysed in this study. The search strategies required to reproduce the record counts reported in Figure 2 are given in full in Section 2.2, and all included studies are listed in Table 1.
